# Therapeutic positioning of secretory acetylated APE1/Ref-1 requirement for suppression of tumor growth in triple-negative breast cancer *in vivo*

**DOI:** 10.1038/s41598-018-27025-9

**Published:** 2018-06-07

**Authors:** Yu Ran Lee, Myoung Soo Park, Hee Kyoung Joo, Ki Mo Kim, Jeryong Kim, Byeong Hwa Jeon, Sunga Choi

**Affiliations:** 1 Research Institute of Medical Sciences, Department of Physiology, School of Medicine, Chungnam National University, Daejeon, 35015 South Korea; 2 Preclinical Research Center, Chungnam National University Hospital, Daejeon, 35015 South Korea; 3 Herbal Medicine Research Division, Korea Institute of Oriental Medicine (KIOM), Daejeon, 34054 South Korea; 4 Department of Surgery, School of Medicine, Chungnam National University, Daejeon, 35015 South Korea

## Abstract

Triple-negative breast cancer (TNBC) represents a relatively small proportion of all BCs but a relatively large proportion of BC-related death. Thus, more effective therapeutic strategies are needed for the management of TNBC. We demonstrated that the stimulation of apoptosis by the binding of secreted acetylated-apurinic apyrimidinic endonuclease 1/redox factor-1 (Ac-APE1/Ref-1) to the receptor for advanced glycation end products (RAGE) was essential for TNBC cell death in response to hyperacetylation. The aim of the present study was to assess the potential therapeutic efficacy of secretory Ac-APE1/Ref-1 in orthotopic TNBC xenografts *in vivo*. We found that hyperacetylation in xenografts caused secretion of Ac-APE1/Ref-1 into the blood, where the factor bound directly to RAGE in hyperacetylated tumor tissues. Hyperacetylation in the TNBC xenografts induced strong inhibition of tumor growth and development, leading to apoptotic cell death, accompanied by increased RAGE expression and generation of reactive oxygen species. Tissues exhibited markedly higher counts of apoptotic bodies, a reduced proliferation index, and reduced neovascularization compared with control tumors. Ac-APE1/Ref-1-stimulated apoptosis was markedly reduced in RAGE-knockdown tumors compared with RAGE-overexpressing tumors, even in the presence of hyperacetylation. The function of secreted Ac-APE1/Ref-1 was confirmed in other hyperacetylated TNBCs xenografts using BT-549 and MDA-MB-468 cells, demonstrating its relevance as an anti-cancer molecule.

## Introduction

Breast cancer is the most common cancer in women worldwide^1^. Triple-negative breast cancer (TNBC) comprises approximately 15% of all breast cancers and exhibits a higher histological grade, higher metastatic occurrence, and earlier recurrence than other breast cancer types, resulting in poor prognoses^2–4^. With the lack of targetable receptors, only systemic chemotherapy is available for treating TNBC patients; however, some cancer cells develop resistance to chemotherapy^5^.

Recent reports, including our own, showed that post-translational modifications (PTMs) play a decisive role in various drug responses^6–11^. PTM of cellular proteins caused by chemotherapeutic agents influences the localization, protein-protein interactions, and modulation of specific signaling cascades and protein expression. We recently demonstrated that co-treatment with the acetyl-group donor, acetylsalicylic acid (ASA) and the deacetylase inhibitor, trichostatin A (TSA) resulted in hyperacetylation in TNBC cells. We also showed that stimulation by secretory acetylated-apurinic apyrimidinic endonuclease 1/redox factor-1 (Ac-APE1/Ref-1) binding to up-regulated receptor for advanced glycation end products (RAGE) was essential for tumor cell death^12^. Based on these observations, a combination of therapeutics promoting both PTMs and apoptosis could potentially improve TNBC therapy.

APE1/Ref-1 is a multifunctional protein involved in DNA repair that controls transcription factor activity based on its redox status. The expression status of this protein is altered in numerous cancers, including prostate, lung, colon, and ovarian tumors^13,14^, and elevated APE1/Ref-1 levels in cancer cells have been targeted to increase susceptibility to both radiation and chemotherapy *in vivo* and *in vitro*^15,16^. In a previous study, we showed that APE1/Ref-1 was acetylated and secreted from TSA-treated HEK-293cells, suggesting extracellular roles^17^. The secretion of high-mobility group box 1 (HMGB1), another intracellular protein expressed by macrophages, is also mediated by acetylation, and prevents nuclear reentry and enables packaging into secretory vesicles^18^. Secreted HMGB1 shows anti-tumor activity as a chemoattractant by activating innate immunity^19^. The transmembrane receptor RAGE binds a broad range of ligands, including advanced glycation end products, amyloid β-peptide, HMGB-l, S100/calgranulins and Ac-APE1/Ref-1, by recognizing ligand patterns, such as acetyl moieties, rather than a unique amino acids sequences, indicating that different cellular responses are transduced through the stimulation of RAGE by various environmental signals^12,20^. For example, in apoptotic signaling, RAGE binds HMGB-1 in neuronal cells and induces cell death via activation of p-38 mitogen-activated protein kinase (MAPK)/ extracellular signal-related kinase (ERK)^21^. Binding of RAGE to S100A8/A9 enhances the activity of natural killer cells and suppresses tumor growth *in vivo*^22^. These findings are consistent with extracellular Ac-APE1/Ref-1 promoting cell death through RAGE in response to hyperacetylation, leading to inhibition of cancer cell proliferation and induction of apoptosis, and not via downregulation of APE1/Ref-1.

Because hyperacetylation induced increased RAGE expression and apoptosis mediated by autocrine and/or paracrine mechanisms involving secreted Ac-APE1/Ref-1 in TNBC cells^12^, we hypothesized that secreted Ac-APE1/Ref-1 could promote apoptosis in hyperacetylated TNBC xenografts *in vivo*. We tested this hypothesis in xenograft models using three human basal subtyped TNBC cell lines (MDA-MB-231, BT-549, and MDA-MB-468).

## Results

### Ac-APE1/Ref-1 secreted in response to hyperacetylation directly binds RAGE in tumors

**Figure 1:**
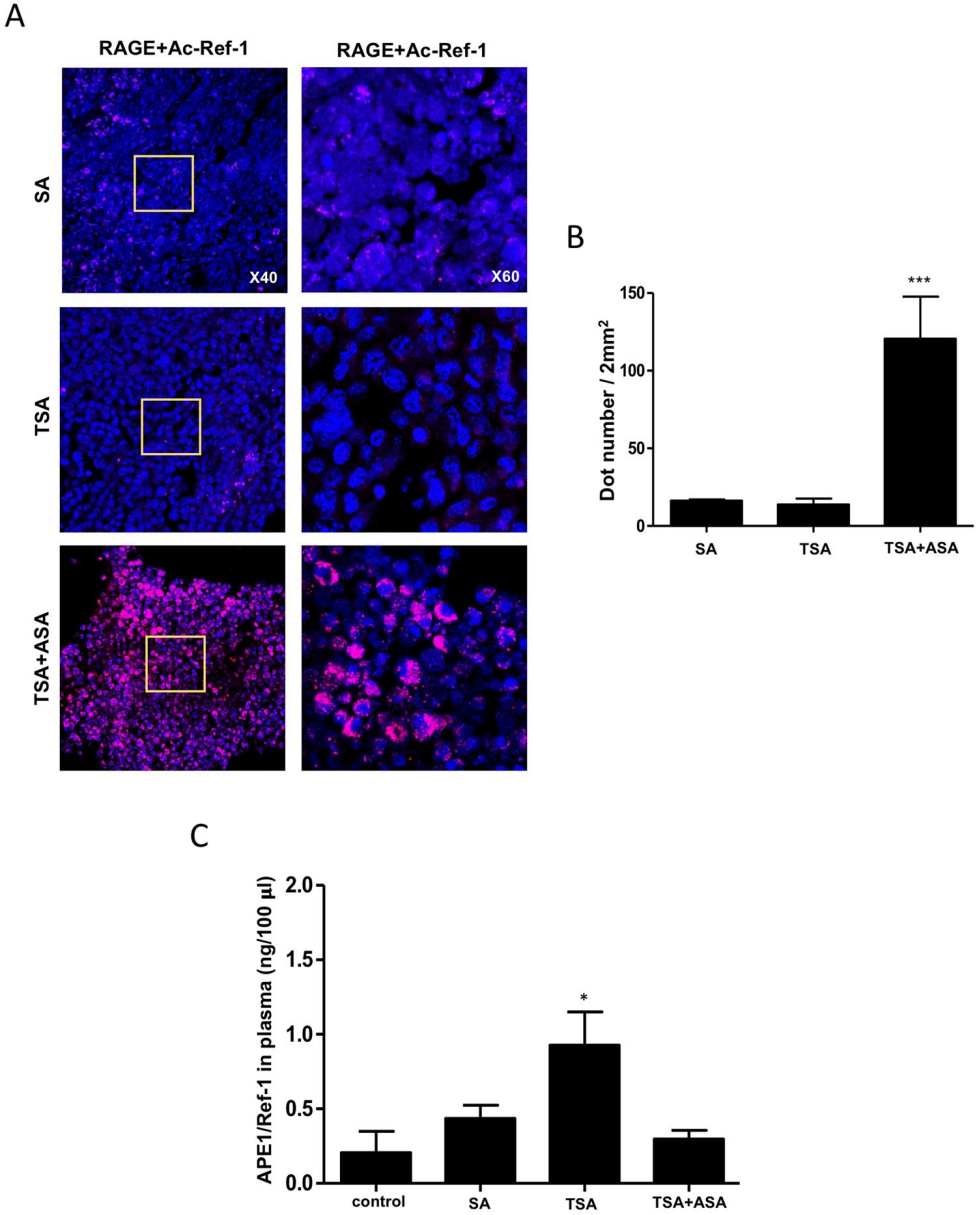
Ac-APE1/Ref-1 is secreted in response to hyperacetylation and subsequently binds to RAGE. (**A**) MDA-MB-231 xenografts were treated with 20 mg/(kg·day) ASA plus subcutaneous injection of 0.5 mg/kg TSA, TSA alone, or SA (as a negative control) three times weekly, and the tumor sections from each xenograft were analyzed by PLA. PLA-specific red fluorescence, representing binding signals between RAGE and secreted Ac-APE1/Ref-1 in hyperacetylated tumor sections, was observed using 3D confocal microscope. (**B**) Numbers of red dots on cells (*n* > 100) counted in five randomly selected areas (2 mm^2^) in each experiment. (**C**) Plasma levels of APE1/Ref-1 was measured by ELISA. Columns represent the mean (*n* = 5); bars represent the SE. *P < 0.05, ***P < 0.001 versus SA-treated control cells as determined by one-way ANOVA followed by Dunnett’s test. Similar results were observed in triplicate experiments.

We previously suggested that hyperacetylation of TNBC cells resulted in cell death through the action of secreted Ac-APE1/Ref-1, which functioned as an apoptotic trigger^12^. Here, we studied the role of secreted Ac-APE1/Ref-1 in tumors from hyperacetylated xenografts. First, we determined whether secreted Ac-APE1/Ref-1 directly binds RAGE in tumors using PLA (proximity ligation assay). Secreted Ac-APE1/Ref-1 binding to TNBC tumor sections was visible as numerous pink spots (Fig. 1A), whereas treatment with salicylic acid (SA) without the acetyl moiety or TSA yielded only faint background signals. Hyperacetylated tumor sections exhibited a 6.7-fold stronger signal, indicating a proximity of <40 nm between RAGE and Ac-APE1/Ref-1 versus the SA- or TSA-treated tumor sections (Fig. 1B).

Secretory APE1/Ref-1 in plasma from xenografts was quantified by ELISA^17,23^. Remarkably, the levels of secreted APE1/Ref-1 were ~2.3-fold lower in hyperacetylated xenografts (0.35 ng/100 μl) than in xenografts from TSA-only-treated mice (0.81 ng/100 μl) but comparable to the levels in SA-treated xenografts (0.41 ng/100 μl) (Fig. 1C). These results demonstrate that secreted Ac-APE1/Ref-1 directly binds to RAGE in response to hyperacetylation, indicating a functional extracellular role of Ac-APE1/Ref-1 *in vivo*.

### Hyperacetylation inhibits the growth of MDA-MB-231 orthotopic xenografts

**Figure 2:**
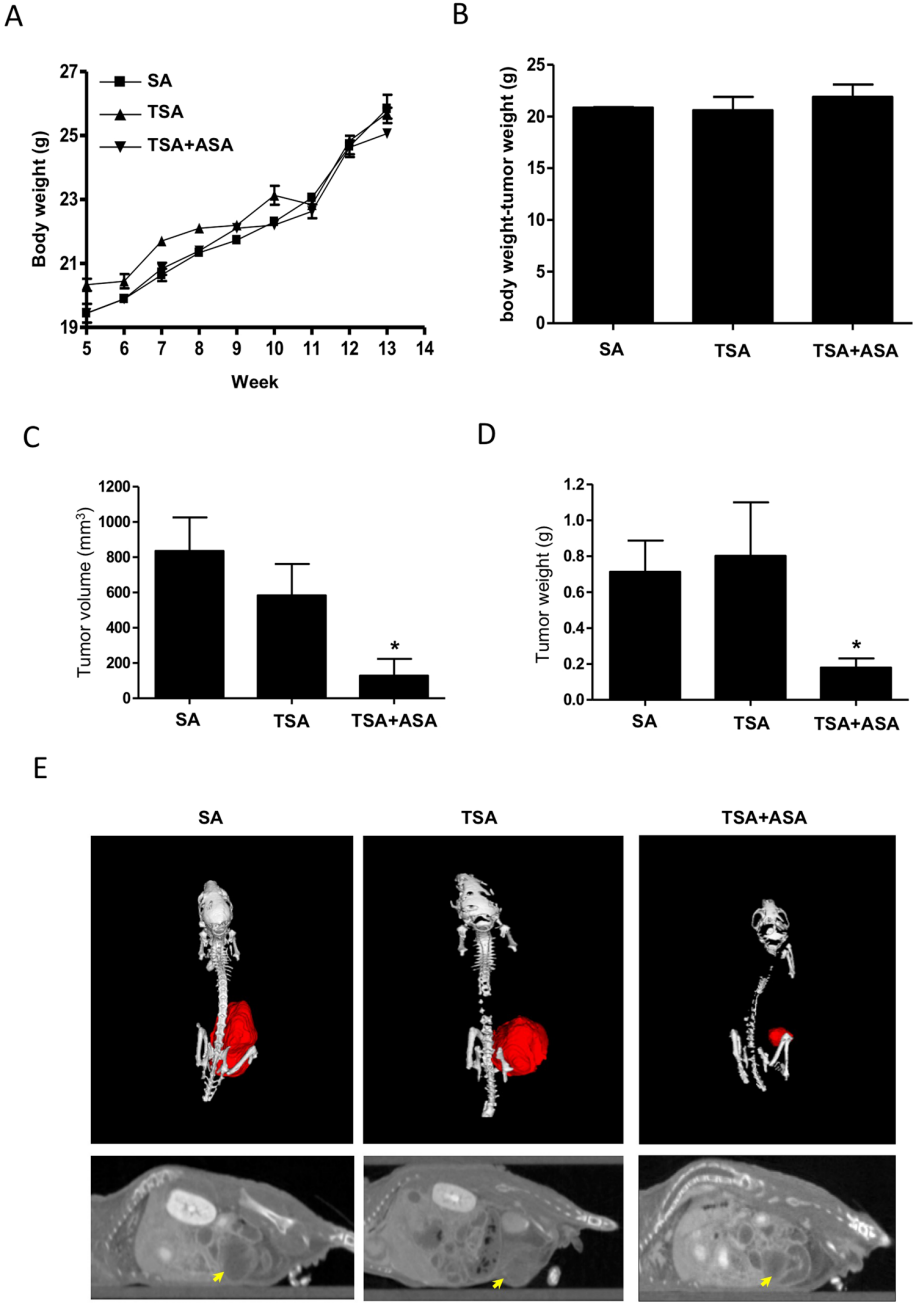
Hyperacetylation suppresses growth of MDA-MB-231 cells implanted in female nude mice. (**A**–**D**) Effect of oral administration of 20 mg/(kg·day) ASA plus subcutaneous injection of 0.5 mg/kg TSA three times weekly on total body weight (A), body weight in the absence of tumors (**B**), tumor volume, (**C**) and wet-tumor weight (**D**). Data reflect the mean (*n* = 6–7 mice/group, with tumor cells orthotopically injected into the mammary fat pad of each mouse). *P < 0.05 versus control mice according to one-way ANOVA, followed by Bonferroni’s test. (**E**) Representative micro-CT of xenografts treated with 20 mg/(kg·day) + 0.5 mg/kg TSA, TSA alone or SA.

We determined whether hyperacetylation inhibits tumor growth of MDA-MB-231 xenografts, and whether it causes cell death by activating apoptosis-related proteins in tumors. We observed the effect of hyperacetylation on MDA-MB-231 xenograft tumor growth through ASA treatment by oral gavage plus subcutaneous TSA injection three times per week. To prevent necrotic cell death, a lower concentration of ASA or TSA was selected than that used in previous studies^24–26^. The average body weights of the control and hyperacetylated mice did not differ significantly throughout the experiment, and no other signs of distress, such as impaired movement and posture, indigestion, or areas of redness or swelling, were observed (Fig. 2A). The average body weights of the hyperacetylated mice without tumors were higher albeit not significantly, than those of SA- or TSA-treated mice (Fig. 2B). These data indicate normal growth in hyperacetylated mice. Additionally, the average tumor volume and endpoint weight in hyperacetylated mice were significantly lower than those in TSA-treated mice (Fig. 2C,D). The average volume and wet weight of tumors in hyperacetylated mice (128.8 mm^3^ and 0.179 g, respectively) were ~4.2-fold lower than those of TSA-only-treated xenografts (*P* < 0.05; TSA vs. hyperacetylated mice; Fig. 2C,D). Microcomputed (micro-CT) imaging showed well-delineated tumors that were suitable for visual volumetric analysis after intravenous contrast-agent administration. Hyperacetylated xenografts produced smaller, red tumors than those in SA- or TSA-treated mice, which agreed with the measured tumor weights (Fig. 2E). The smallest detectable tumor measured by micro-CT of a hyperacetylated xenograft was 200 µm, and the mean tumor diameter (X × Y) was 43.70 ± 13.02 mm, whereas the mean tumor volume was 128.8 ± 94.7 mm^3^. These results indicate that hyperacetylation significantly inhibited MDA-MB-231 xenograft growth without apparent side effects.

### Attenuated tumor cell growth causes apoptosis in tumors of hyperacetylated mice

**Figure 3:**
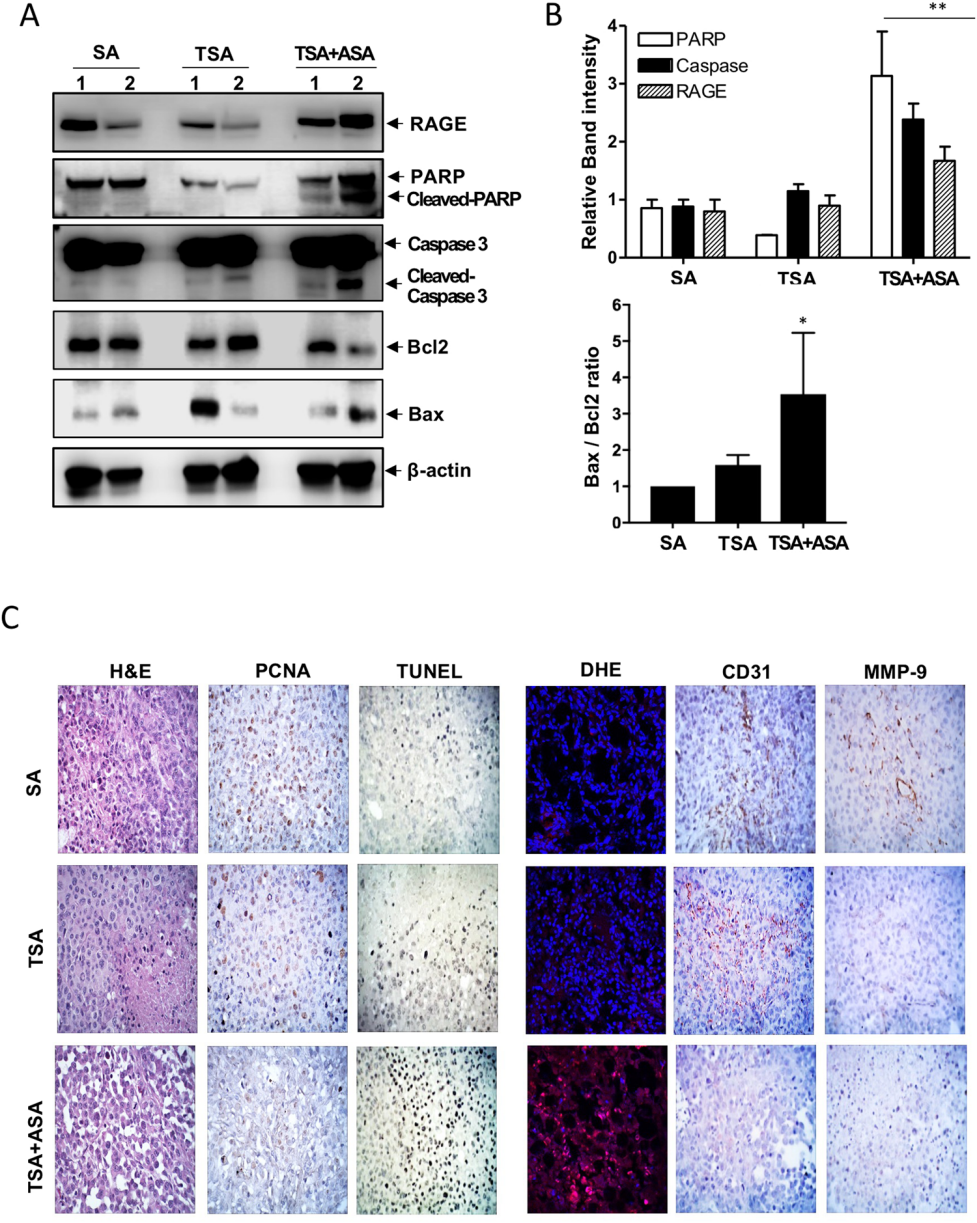
Hyperacetylation induces apoptosis in MDA-MB-231 cell xenografts, causing upregulation of RAGE. Immunological analysis of the tumor tissues from MDA-MB-231 xenografts treated with 20 mg/(kg·day) ASA plus subcutaneous injection of 0.5 mg/kg TSA, TSA alone or SA (as a negative control) three times weekly. Numbers on the top of band present individual tumors which were derived from different xenografts. (**A** and **B**) Representative immunoblots showing RAGE, Bax, Bcl-2, and caspase-3 and PARP-1 expression. β-actin was used as a protein-loading control. Immunoblotting was performed two or more times for each protein by using independently prepared lysates with similar results. Full-length blots are presented in Supplementary Fig. S3. Fold changes in the levels of apoptosis markers relative to the control are shown for each treatment. Columns represent the mean (*n* = 3); bars represent the SE. *P < 0.05, **P < 0.01 versus SA-treated control cells as determined by one-way ANOVA followed by Dunnett’s test. Representative results of replicate experiments with similar results are shown. (**C**) Representative staining images for histology (H&E staining), proliferation index (PCNA staining), neovascularization (CD31 staining), metastatic index (MMP-9 staining), apoptotic bodies (TUNEL staining) and ROS generation (DHE staining) in tumor sections from three mice in each group.

RAGE was activated with Ac-APE1/Ref-1 to initiate intracellular signaling (Fig. 1), and the effects of hyperacetylation on apoptosis-related proteins in tumors were determined. Hyperacetylation of MDA-MB-231 xenografts resulted in a significant increase in RAGE expression compared with tumors from SA- or TSA-treated xenografts (Fig. 3A,B). Hyperacetylation also caused a marked increase in the Bax/Bcl-2 ratio along with PARP-1 cleavage following caspase-3 activation, although cleaved p17 of caspase-3 was only detected in TSA-treated xenografts (Fig. 3A,B). Together, these results confirmed that tumor hyperacetylation caused apoptosis through a RAGE-mediated signaling pathway initiated by binding of Ac-APE1/Ref-1 and alteration of Bcl-2 expression, leading to caspase activation and PARP-1 cleavage.

The alteration of cell proliferation and apoptosis induction was confirmed by histological findings. Tumor sections from SA- and TSA-treated and hyperacetylated mice were stained with H&E, which revealed a compact epithelial cell mass in xenografts of mice treated with SA or TSA (Fig. 3C, *left*), whereas tumors from hyperacetylated mice appeared as loose epithelial cells with scattered apoptotic cells characterized by a dark, shrunken cytoplasm and purple, pyknotic nuclei. SA- or TSA-treated tumors displayed higher proliferation indexes than tumors from hyperacetylated mice, as indicated by PCNA staining. Furthermore, tumors from hyperacetylated mice exhibited markedly higher counts of apoptotic bodies than SA- or TSA-treated tumors, as indicated by TUNEL staining (Fig. 3C, *left*).

Inhibition of eukaryotic cell proliferation resulting in cell death involves the generation of ROS, which can stimulate the expression of cell death signaling proteins^27^. We hypothesized that intracellular signaling following RAGE stimulation leads to ROS generation, thereby promoting hyperacetylation-mediated inhibition of cell proliferation and induction of apoptosis. To test this hypothesis, we determined the effect of hyperacetylation on ROS generation in fresh-frozen-tumor sections. Notably, DHE staining indicated that ROS generation, visualized as a bright pink color in hyperacetylated tumor tissue, likely resulted from a RAGE signal transduction cascade, as SA- and TSA-treated tumor tissues exhibiting no changes in RAGE expression were not ROS-positive (Fig. 3C, *right*). To determine the effect of hyperacetylation on neovascularization and metastatic potential, tumor sections were stained for the endothelial cell marker CD31 and the invasion marker MMP-9 (Fig. 3C, *right*). Tumors from hyperacetylated mice exhibited fewer morphological changes and CD31-positive blood vessels as well as lower MMP-9 expression than SA- and TSA-treated tumors. Metastatic tumors in abdominal/lung tissue were identified in two to three mice from the SA- or TSA-treated groups at the experimental endpoint. These results indicated that hyperacetylation-mediated suppression of MDA-MB-231 xenograft growth *in vivo* was associated with increased apoptosis and decreased proliferation, neovascularization, and invasiveness. Furthermore, the anti-tumor effects were induced through binding of secreted Ac-APE1/Ref-1 to RAGE, which caused ROS generation, increased Bax/Bcl-2 ratio, and caspase activation.

### Hyperacetylation-induced apoptosis depends on Ac-APE1/Ref-1-bound RAGE

**Figure 4:**
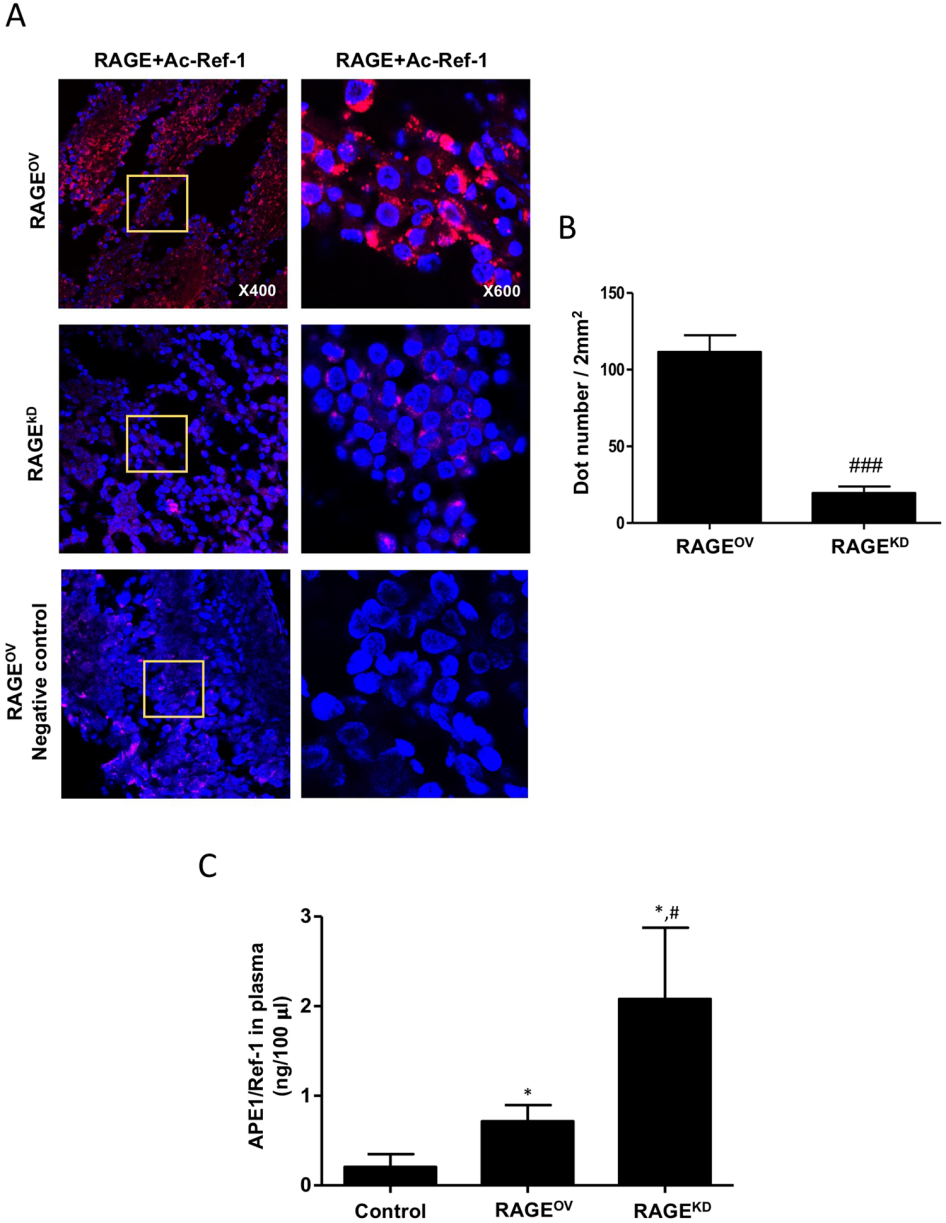
Secreted Ac-APE1/Ref-1 binds to RAGE in RAGE^OV^ but not in RAGE^KD^ mice. (**A**) Xenografts implanted with MDA-MB-231 cells overexpressing RAGE (RAGE^OV^) or with knocked-down RAGE expression (RAGE^KD^) were treated three times weekly with 20 mg/(kg·day) ASA plus subcutaneous injection of 0.5 mg/kg TSA, TSA alone, or SA (as a negative control), and tumor sections from each xenograft were analyzed by PLA. PLA-specific red fluorescence representing binding signals associated with RAGE binding to secreted Ac-APE1/Ref-1 in hyperacetylated tumor sections was observed by 3D confocal microscopy. (**B**) The number of red dots on cells (*n* > 100) was counted in five randomly selected areas (2 mm^2^) in each experiment. (**C**) Plasma levels of APE1/Ref-1 as measured by ELISA. Columns represent the mean (*n* = 5); bars represent the SE. ^#^*P* < 0.05, ^###^*P* < 0.001 versus RAGE^OV^ cells according to an unpaired *t-*test, *P < 0.05 versus SA-treated control mice as determined by one-way ANOVA followed by Dunnett’s test. Similar results were observed in triplicate experiments.

RAGE is a transmembrane receptor that is activated by various ligands, leading to differential intracellular signaling^20^. Activated RAGE and p-38 MAPK/ERK mediate apoptotic signaling, which regulates cancer cell proliferation^21^. Given that hyperacetylation increases RAGE expression in tumors, we hypothesized that RAGE stimulation via Ac-APE1/Ref-1 binding could sensitize or prime tumor cells for apoptosis *in vivo*. We previously investigated the ability of RAGE to trigger intracellular signaling and influence tumor growth in hyperacetylated xenografts using stable overexpression (RAGE^OV^) or knockdown (RAGE^KD^) of RAGE^12^. As expected, hyperacetylation in RAGE^OV^ tumors promoted binding of Ac-APE1/Ref-1 to RAGE (Fig. 4A). Tumor sections from hyperacetylated RAGE^OV^ xenografts showed numerous fluorescent interaction signals, whereas hyperacetylated tumors from RAGE^KD^ xenografts showed weak signals, with signals from RAGE^OV^ xenografts that were 5.5-fold stronger than those from RAGE^KD^ xenografts, confirming Ac-APE1/Ref-1–RAGE binding (Fig. 4B). Additionally, the level of secretory Ac-APE1/Ref-1 in the plasma was ~3.86-fold lower in RAGE^OV^ (0.7 ng/100 μl) than in RAGE^KD^ (2.7 ng/100 μl) xenografts, although all xenografts were hyperacetylated (Fig. 4C). These results demonstrated that RAGE stimulation by direct binding of secreted Ac-APE1/Ref-1 could initiate intracellular signaling in hyperacetylated tumors.

**Figure 5:**
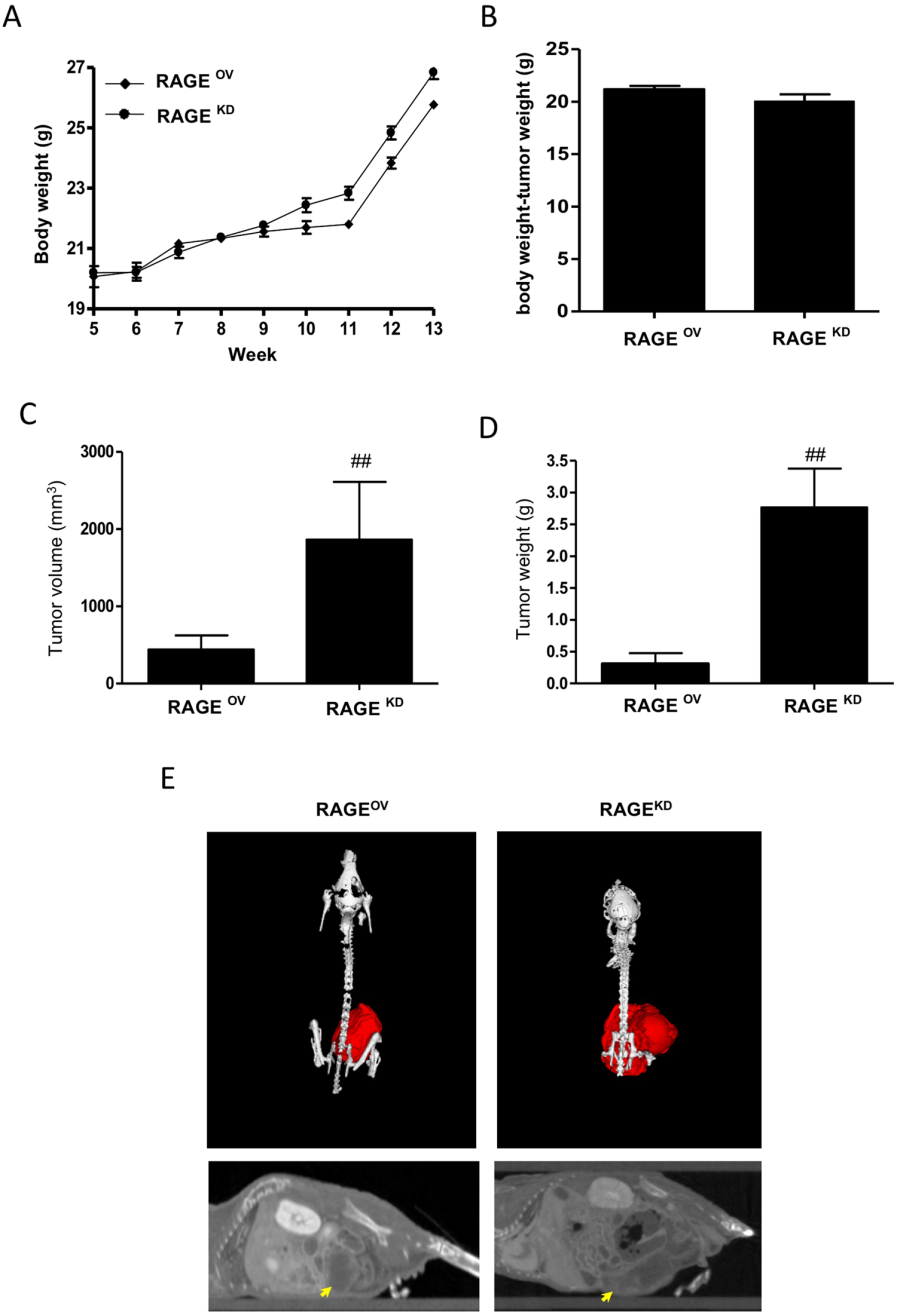
Hyperacetylation attenuates tumor growth in RAGE^OV^, but not in RAGE^KD^ xenografts. (**A**–**D**) Effect of oral administration of 20 mg/(kg·day) ASA plus subcutaneous injection of 0.5 mg/kg TSA three times weekly on total body weight (A), body weight in the absence of tumors (**B**), tumor volume (**C**), and tumor wet weight (**D**). Data points shown represent the mean (*n* = 6–7 mice/group with tumor cells orthotopically injected into the mammary fat pad of each mouse). ^##^*P* < 0.01 versus RAGE^OV^ cells according to an unpaired *t-*test. (**E**) Representative micro-CT of RAGE^OV^ and RAGE^KD^ xenografts that were treated with 20 mg/(kg·day) + 0.5 mg/kg TSA, TSA alone, or SA.

To determine the effects of RAGE stimulation through hyperacetylation in xenograft tumors, we monitored body weight in mice carrying hyperacetylated xenografts derived from RAGE^OV^ or RAGE^KD^ cells. No significant difference was observed in body weight according to tumor type at the experimental endpoint, although a modest increase in average body weight of RAGE^KD^ mice resulted from their larger tumors. Mice with hyperacetylated xenografts from RAGE^OV^ cells showed normal body weight gain with aging, comparable to that in mice without tumors (Fig. 5A,B). However, the average tumor volume and weight of RAGE^OV^-derived xenografts were significantly smaller than those of RAGE^KD^-derived xenografts, with an average tumor volume in RAGE^OV^ mice (403 mm^3^) ~4.62-fold lower than that in RAGE^KD^ mice (1,863 mm^3^). Consistently, the average tumor weight in RAGE^OV^ mice (0.307 g) was ~9-fold lower than that in RAGE^KD^ mice (2.766 g) (*P* < 0.05; Fig. 5C,D). Hyperacetylated RAGE^OV^ mice showed smaller, red tumors compared with hyperacetylated RAGE^KD^ mice (Fig. 5). These results demonstrated RAGE involvements in effectively attenuating tumor growth in hyperacetylated mice and supported a role for secreted Ac-APE1/Ref-1 in mediating this effect.

**Figure 6:**
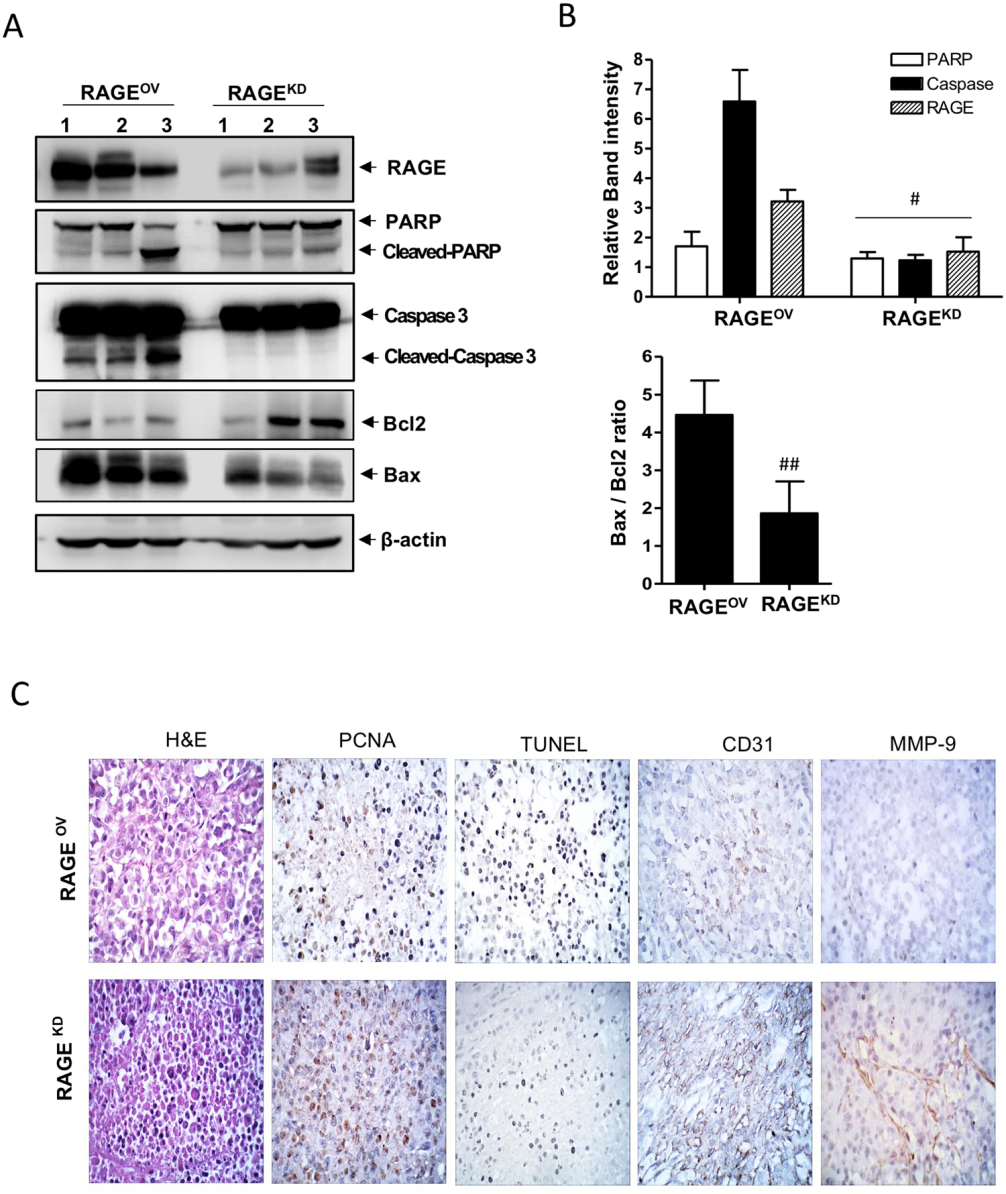
Hyperacetylation induces apoptosis in RAGE^OV^ but not in RAGE^KD^ xenografts. Immunological analysis of the tumor tissues from RAGE^OV^ and RAGE^KD^ xenografts treated with 20 mg/(kg·day) ASA plus subcutaneous injection of 0.5 mg/kg TSA three times weekly. Numbers on the top of the band present individual tumor which were derived from different xenografts. (**A** and **B**) Representative immunoblots for RAGE, Bax, Bcl-2, caspase-3, and PARP-1. β-actin was used as a protein-loading control. Immunoblotting for each protein was performed at least twice using independently prepared lysates with similar results. Full-length blots are presented in Supplementary Fig. S4. Fold changes in apoptosis marker levels relative to the control are shown for each treatment. Columns represent the mean (*n* = 3); bars represent the SE. ^#^*P* < 0.05, ^##^*P* < 0.01 versus RAGE^OV^ cells was determined by one-way ANOVA followed by Bonferroni’s test. Data are representative of replicate experiments with similar results. (**C**) Representative staining images for histology (H&E staining), proliferation index (PCNA staining), neovascularization (CD31 staining), metastatic index (MMP-9 staining), and apoptotic bodies (TUNEL staining) from tumor sections from three mice of each group.

We confirmed that the apoptotic signaling in hyperacetylated tumors of RAGE^OV^ mice was also attributable to growth-inhibitory effects. Hyperacetylated tumors of RAGE^OV^ mice exhibited PARP-1 cleavage and higher RAGE expression and caspase-3 activation, resulting in an increased Bax/Bcl-2 ratio, as compared to tumors from RAGE^KD^ hyperacetylated xenografts (Fig. 6A,B). Additionally, TUNEL staining demonstrated the induction of apoptosis (Fig. 6C), and tumor sections from hyperacetylated RAGE^OV^ mice exhibited markedly more apoptotic bodies than those from hyperacetylated RAGE^KD^ mice. H&E-based pathological examination of tumor sections from hyperacetylated RAGE^OV^ mice showed more differentiated cell morphology, with a low-to-moderate nucleus-to-cytoplasm (N:C) ratio, whereas RAGE^KD^ tumors exhibited a small cytoplasm and darkly stained nuclei with very high N:C ratios, even after hyperacetylation. These results were closely related to the regulation of cell proliferation as shown by PCNA staining. Tumors from hyperacetylated mice with RAGE^KD^-derived xenografts had relatively higher proliferation indexes than those of hyperacetylated RAGE^OV^ mice (Fig. 6C). Immunohistochemical staining for CD31 and MMP-9 showed that tumor cell neovascularization and invasiveness in hyperacetylated tumors were lower in RAGE^OV^ than in RAGE^KD^ mice. Notably, nearby breast and lung metastases were identified in two RAGE^KD^ mice on the termination day, even after hyperacetylation treatment. These results reinforced that hyperacetylation-mediated regulation of tumor growth, as well as neovascularization and invasive activities, promoted apoptosis via RAGE-mediated signaling as a major pathway, suggesting the potential of Ac-APE1/Ref-1 as an apoptotic trigger.

### Hyperacetylation therapy in TNBC-xenografted mice inhibits tumor growth and prevents spontaneous metastases

**Figure 7:**
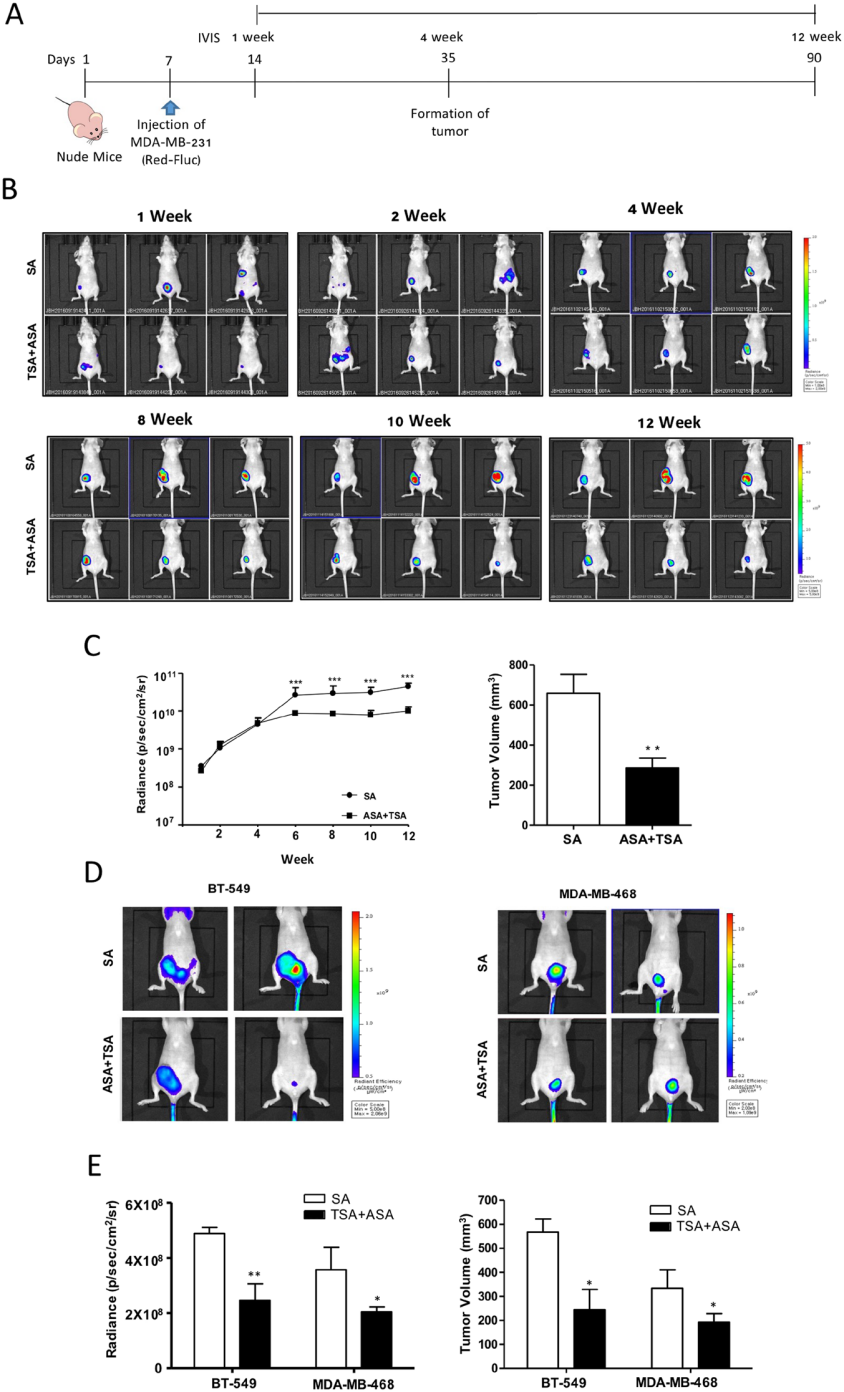
Hyperacetylation significantly reduces tumor growth *in vivo*. (**A**–**C**) Schematic representation of the treatment regime for the administration of 20 mg/(kg·day) ASA plus subcutaneous injection of 0.5 mg/kg TSA, or SA (as a negative control) three times weekly in MDA-MB-231 cells xenografts. Female nude mice were injected with 1.5 × 10^6^ MDA-MB-231-FLuc cells. After the formation of detectable tumors according to IVIS imaging (7 days post injection), mice were weighed and randomly assigned to SA or ASA + TSA groups (*n* = 7, animals/group) (**A**). Representative IVIS images obtained after luciferin injection at different days post-injection of MDA-MB-231-FLuc cells, showing reduced tumor growth in hyperacetylated mice (**B**). Tumor-growth curves and tumor weights for both animal groups are shown for up to 90 days. Based on a slope comparison test, the treated animals showed a marked reduction in tumor growth [t (6) = 2.6]. ***P < 0.001 versus SA-treated control mice by an unpaired *t*-test (**C**). (**D** and **E**) Female nude mice were injected with 1.5 × 10^6^ BT-549 or MDA-MB-468 cells. After the formation of detectable tumors according to IVIS imaging with MMPSense750, the mice were weighed and randomly assigned to SA or ASA + TSA group (*n* = 4 animals/group). Tumor growth and tumor volume in both animal groups were assessed for up to 60 days. Columns represent the mean (*n* = 4); bars represent the SE. ^*^P < 0.05; ^**^P < 0.01 versus SA-treated control mice as determined by an unpaired *t-*test.

Having shown the anti-tumor effects of hyperacetylation by micro-CT imaging at the experimental endpoint, we determined the real-time effects of hyperacetylation using IVIS in TNBC xenografts generated using three different cell lines. MDA-MB231-FLuc, MDA-MB-468 cells, and BT-549 cells were detected after injection of luminescent luciferin or a fluorescent MMPSense 750 probe, respectively. Using whole-body IVIS, we monitored tumor development for 12 weeks starting one week post tumor-cell implantation (Fig. 7A). Luminescent MDA-MB-231 tumors were visible through the skin as early as 7 days post-implantation. During the 90-day real-time observation period, the tumor growth rate decreased in hyperacetylated mice as compared to SA-treated mice according to the relative luminescence intensities (Fig. 7B). In accordance with the marked decrease in tumor volume as recorded by caliper measurements, a major reduction in the amount of luminescent tumor cells was observed from days 24 to 90, resulting in a 4-fold decrease in the mean luminescent tumor area of tumors in hyperacetylated mice (Fig. 7C). Interestingly, a significant difference was observed earlier, reflecting the higher accuracy of the bioluminescent IVIS relative to the measured values near the experimental endpoint. Hyperacetylation-mediated regulation of tumor growth was found in two additional TNBC xenografts using biofluorescent IVIS (Fig. 7D). Additionally, hyperacetylation suppressed tumor growth according to the significant decrease in tumor biofluorescence intensities in both BT-549 and MDA-MB-468 xenografts. Compared with SA-treated mice, tumors from hyperacetylated MDA-MB-468 and BT-549 xenografts were significantly smaller (2.1- and 1.8-fold decrease, respectively) (Fig. 7E). During the experimental period, no noticeable metastasis occurred in the breast or other organs based on biosignal intensity monitoring. Collectively, these data suggested that hyperacetylation therapy inhibited tumor growth and spontaneous metastases.

### Recombinant Ac-APE1/Ref-1 induces apoptotic cell death in hyperacetylated TNBC cells, but not in normal cells

**Figure 8:**
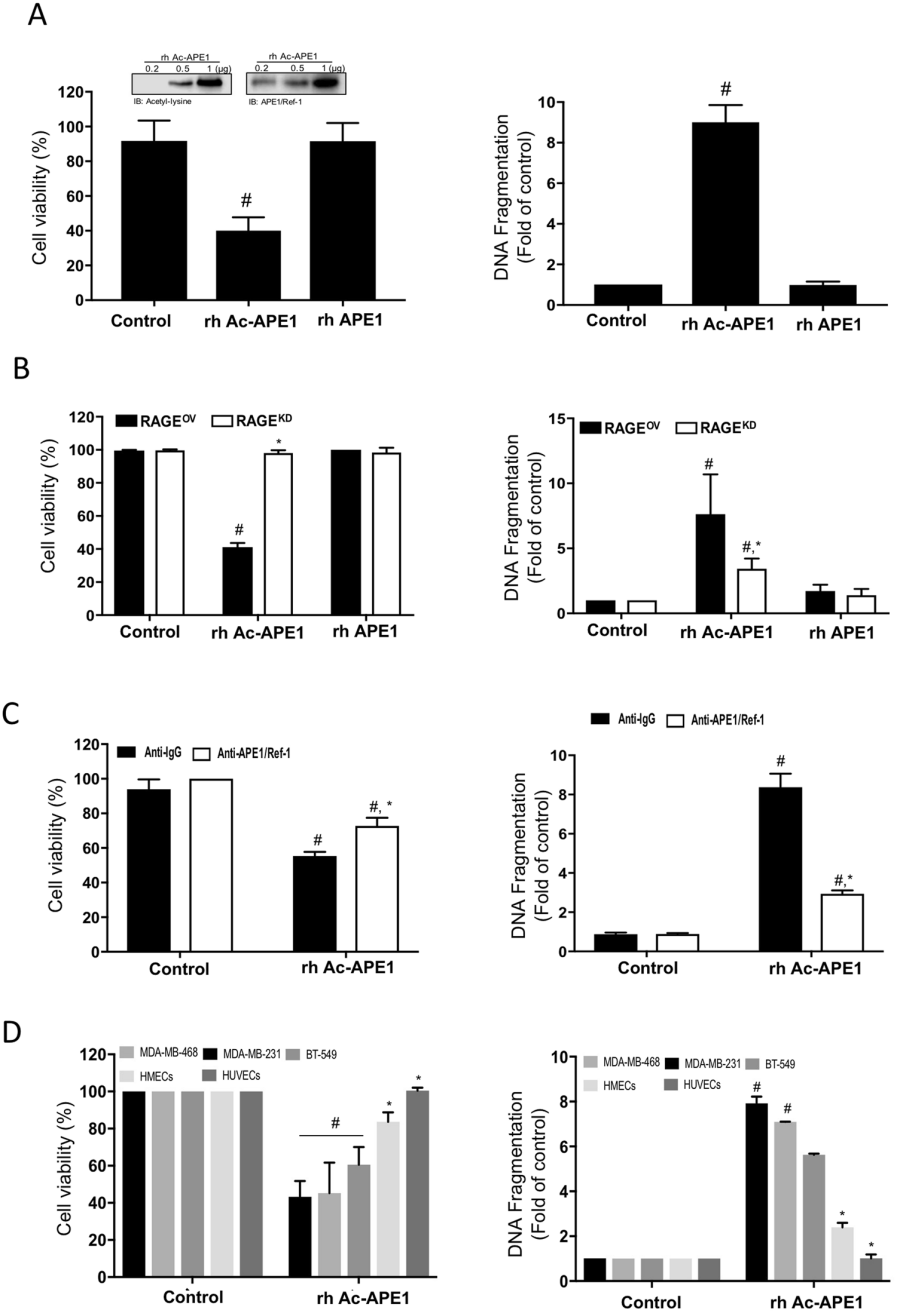
Recombinant Ac-APE1/Ref-1 induced apoptotic cell death by binding to RAGE in TNBCs, leading to a decrease in cell viability, but not in normal cells. (**A** and **C**) Wild-type MDA-MB-231, (**B**) RAGE^OV^, or RAGE^KD^ MDA-MB-231 cells (**D**) MDA-MB-231, MDA-MB-468, BT-549 cells, HMECs, and HUVECs were treated with 5 mM ASA in the presence of 0.1 μM TSA. After incubation for 6 h, the medium was replaced with fresh medium. Cells were further treated with rh Ac-APE1/Ref-1 (1 µg) or rh APE1/Ref-1 (1 µg) for 24 h. The inset shows acetylated- or native form of APE1/Ref-1. Cell viability and cytoplasmic histone-associated DNA fragmentation were determined. (**C**) Blocking effect of the anti-APE1/Ref-1 antibody on the binding interaction between RAGE and Ac-APE1/Ref-1. Cells where the medium was replaced with fresh medium were pretreated with rabbit-IgG (2 µg) or anti-APE1/Ref-1 (2 µg) for 2 h and then further incubated with rh Ac-APE1/Ref-1. (**B)** to (**C)**, *columns*, mean (n = 3); *bars*, SE. *P < 0.05 versus between groups by one-way ANOVA followed by Bonferroni’s multiple comparison test. *Columns*, mean (n = 3); *bars*, SE. *P < 0.05 versus within-group by one-way ANOVA followed by Bonferroni’s multiple comparison test. In (**A**) to (**D**), *columns*, mean (n = 3); *bars*, SE. ^#^*P* < 0.01 versus control cells was determined by one-way ANOVA followed by Bonferroni’s test.

To confirm the possibility of Ac-APE1/Ref-1 as an anti-TNBC therapeutic agent, TNBC cells were examined for evidence of apoptotic cell death using recombinant human Ac-APE1/Ref-1 (rh Ac-APE1/Ref-1). Treatment of MDA-MB-231 cells with rh Ac-APE1/Ref-1 markedly decreased cell viability compared to rh APE1/Ref-1-treated cells even in hyperacetylation (Fig. 8A). Consistent with the cell viability results, the cytoplasmic histone-associated DNA fragmentation induced by exposure to rh Ac-APE1/Ref-1 was significantly increased (Fig. 8A). The greater magnitude of the effects of rh Ac-APE1/Ref-1 but not rh APE1/Ref-1 on cell viability and DNA fragmentation in RAGE^OV^ cells than in RAGE^KD^ cells indicates that rh Ac-APE1/Ref-1 has a promising role as a triggering molecule to initiate RAGE-mediated cancer cell death signaling (Fig. 8B). As a key feature of apoptosis, the DNA fragmentation in rh Ac-APE1/Ref-1-stimulated cell death was significantly attenuated by an anti-APE1/Ref-1 antibody. The Ac-APE1/Ref-1-mediated decrease in cell viability was recovered to 72.8% after removal of Ac-APE1/Ref-1, and the level of DNA fragmentation was conversely decreased to 35% of that in anti-IgG treated cells (Fig. 8C). Similar to findings in MDA-MB-231 cells, the treatment with rh Ac-APE1/Ref-1 also caused decreased viability and increased apoptosis in other hyperacetylated MDA-MB-468 and BT-549 cells with up-regulated RAGE (Fig. 8D, Supplementary Fig. 2). Noticeably, any appreciable effects of rh Ac-APE1/Ref-1 on cell viability and DNA fragmentation were not observed in normal cells including human mammary epithelial cells (HMECs), and umblical vein endothelial cells (HUVECs), even in hyperacetylated conditions. These results clearly demonstrate that Ac-APE1/Ref-1 possesses potent chemotherapeutic efficacy against hyperacetylated TNBCs but not normal cells based on stimulation of cell death by RAGE-dependent triggering of Ac-APE1/Ref-1.

## Discussion

Our data reveals for the first time the hyperacetylation-mediated cell death mechanism *in vivo*. Previous reports showed that acetylation caused by treatment with histone-deacetylase inhibitors or acetyl-group donors *per se* induced chemotherapeutic activities, affecting DNA repair, protein stability, cell motility, angiogenesis, and radiosensitivity^9,10,28,29^. In this study, using MDA-MB-231 xenografts, a dose of ASA [20 mg/(kg·day)] or TSA (0.5 mg/kg; three times weekly) was administrated to induce hyperacetylation. These doses used were lower than that used previously (ASA: ~100 mg/kg; TSA: ~2 mg/kg daily) to induce tumor cell death *in vivo*^25,26,30,31^. However, the TSA dose applied in combination with other agents was comparable with that used previously^32,33^. Acetylation of cellular proteins occurring as a result of the ASA + TSA treatment was superior to that observed in SA-treated control cells and that induced by ASA or TSA treatment alone (Supplementary Fig. S1A). Increased apoptosis as a result of RAGE stimulation by Ac-APE1/Ref-1 was noticeable in tumors co-treated with ASA + TSA and according to acetylation as shown by immunoblotting and immunohistochemical staining (Supplementary Fig. S1B). These data agreed with our mechanistic studies showing that secreted Ac-APE1/Ref-1 triggers a RAGE-dependent antitumor effect in response to hyperacetylation^12^. ASA is quickly absorbed and distributed to all tissues, resulting in peak plasma levels within 35 to 40 min in a concentration-dependent manner^34^. The acetyl moieties from ASA covalently bind to other proteins in the blood. ASA + TSA treatment is sufficient to maintain secreted Ac-APE1/Ref-1 status in the blood without loss of the acetyl group. Eventually, the Ac-APE1/Ref-1 binds to its pattern-recognition partner RAGE, activating an intracellular signaling cascade. Consistent with our observations *in vitro*^12^, hyperacetylation effectively inhibited the growth and viability of TNBC *in vivo*.

Hyperacetylation-induced apoptosis was also observed in other TNBC xenografts derived from the MDA-MB-468 and BT-549 cell lines. The suppression of tumor growth and the cell death induction in these hyperacetylated mice were accompanied by altered RAGE expression and PARP-1 cleavage (Supplementary Fig. S2) as shown in whole-body IVIS images in the absence of fluorescent metastatic cells. These results suggest that the mode of death in TNBCs could be uniformly explained by hyperacetylation-mediated apoptosis. This mechanism might involve the secretion of the signaling molecule Ac-APE1/Ref-1, continuous stimulation of signaling through autocrine or paracrine pathways, and upregulation of the receptor, RAGE.

The RAGE signaling cascade can be characterized as a feed-forward loop whereby increased oxidative stress results in outcomes such as increased cell proliferation and cell death. The involvement of RAGE in tumor growth, invasion, metastasis, and angiogenesis, including in breast cancer, has been demonstrated by experimental blockade of RAGE signaling or of RAGE itself in *in vitro* analyses and studies in mouse models^35–37^. In contrast to the concept of RAGE/ligands as a potent inducer of tumorigenesis, RAGE activation results in increased ROS production by stimulating specific signaling cascades. Triggering with S100A14 caused RAGE-dependent apoptosis via the mitochondrial pathway in esophageal squamous cell carcinoma^38^. RAGE engagement by S100A6 in neuroblastoma cells also induced cell death via JNK phosphorylation with ROS generation^39^. In addition, high expression of RAGE in lung tissue was reduced with carcinogenesis, but re-expression of RAGE inhibited cell proliferation^40^, and retarded tumor growth in mice^41^, further demonstrating the tumor-suppressive functions of RAGE.

Our observations of RAGE-mediated cell death *in vitro*^12^ and *in vivo*, were different from those of other reports^35,36^, although the same TNBCs were studied. One possible reason for the different results is that changes in cellular gene regulation caused by treatment with acetylating agents resulted in up-regulation of RAGE. It is also possible that there are multiple modes of RAGE activation by RAGE-binding partners^42^. Carefully designed experiments, including analysis of co- and post-translationally modified proteins in response to hyperacetylation and identification of recruited intracellular signaling molecules, and the binding domain of RAGE are needed to systematically explore these possibilities.

Our findings demonstrated that the inhibitory effects of hyperacetylation on tumor growth in TNBC xenografts were unlikely to be mediated exclusively by induction of nuclear factor (NF)-κB or cyclooxygenase (COX)-2 induction, even though ASA was administrated during the experimental period. Although some studies have provided evidence that COX-2 expression and NF-κB regulation are strongly associated with the benefits of aspirin intake in colorectal and breast cancers^43,44^, some epidemiological studies showed that improved breast cancer survival associated with aspirin use is independent of COX-2 or NF-κB^30,45^. Rather, the hyperacetylation-induced growth-suppression phenotype of tumors observed in this study could be ascribed to activation of intrinsic apoptotic signaling. This possibility is consistent with the present mechanistic studies, which showed that the antitumor effects of hyperacetylation were mediated by RAGE activation after binding of secreted Ac-APE1/Ref-1 as evidenced by ROS-induced activation of apoptotic pathways, including alteration of the Bax/Bcl-2 protein ratio and caspase activation for PARP-1 cleavage. Furthermore, the abundant TUNEL-positive apoptotic cells showing caspase-3 cleavage, and few PCNA-positive cells in tumors from mice treated with the ASA + TSA regimen provided additional support for this possibility. The extracellular activity of Ac-APE1/Ref-1 through RAGE stimulation in response to hyperacetylation may exert chemotherapeutic effects *in vivo*.

Chemotherapy, either alone or in combination with surgery, is currently the only available systemic therapy for TNBC patients. However, although TNBC responds better than other types of breast cancer during initial treatment, TNBC cells have displayed chemotherapy resistance over time^46,47^. New strategies, including the identification of novel drug target receptors, represent an urgent unmet medical need for this patient population^48^. Our observations provide a mechanistic rationale for TNBC treatment using hyperacetylating agents that provoke an intracellular cell death program via RAGE *in vivo*. Our data also highlight the potent chemotherapeutic efficacy of rh Ac-APE1/Ref-1 as a trigger in cell death signaling. Furthermore, these observations shed new light on therapeutic strategies for treating chemotherapy-resistant TNBC patients, who require chronic intake of chemotherapeutics to obtain a therapeutic benefit. As secretory molecules from hyperacetylated cells can be identified by carefully designed studies accounting for secretory pathways, stimulators of physiological secretion and uptake molecules remain under continuous study.

In conclusion, this study provides pre-clinical *in vivo* evidence of the therapeutic efficacy of a novel and clinically viable hyperacetylation regimen for treating TNBC. Our findings illustrate the need for preclinical trials to evaluate the potential therapeutic effects of Ac-APE1/Ref-1 as an adjuvant in TNBC xenografts with increased RAGE expression induced by long-term, low-dose aspirin therapy to amplify intracellular programmed cell-death signaling. Furthermore, they provide an understanding of acetylating agents for chemotherapy sensitization for the evaluation of clinical treatment of drug-resistant cancer cells.

## Materials and Methods

### Cell culture

Human breast adenocarcinoma cell lines (MDA-MB-231, MDA-MB-468, and BT-549), HMECs (MCF-10A) and luciferase-expressing MDA-MB-231 cells (MDA-MB-231-Red-FLuc Bioware® Brite) were obtained from ATCC (Manassas, VA, USA) and PerkinElmer (Street Waltham, MA, USA), respectively. All cell lines were authenticated by short tandem-repeat profiling and cell morphology assessment. BT-549 cells were maintained in DMEM (Gibco, Grand Island, NY, USA) with 10% FBS, and 0.01 mg/ml human recombinant insulin. MDA-MB-468 cells were maintained in L-15 medium (Gibco) with 10% FBS. All MDA-MB-231 cell lines, including MDA-MB231-FLuc, MDA-MB-231-RAGE^OV^ and MDA-MB-231-RAGE^KD^, were cultured in RPMI 1640 medium (Gibco) with 10% FBS^12^. HMECs and HUVECs (Lonza Group Ltd., Walkersville, MD, USA) were cultured in specific epithelial and endothelial growth medium (Lonza), respectively.

### Proximal ligation assay

Binding between RAGE and Ac-APE1/Ref-1 was visualized using a Duolink II fluorescence kit (Sigma-Aldrich, St Louis, MO, USA) as described previously^12^, with some modifications. Sections were first incubated with a blocking solution, followed by an overnight incubation with a primary anti-RAGE antibody (clone A11; Santa Cruz Biotechnology, Dallas, TX, USA) and an anti-APE1/Ref-1 antibody. To determine *in situ* whether the proteins co-localized within a 40-nm distance, conjugated oligonucleotides [anti-mouse antibody (minus strand) and anti-rabbit antibody (plus strand)] were added and incubated for 1 h at 37 °C. A negative control was instituted by adding only anti-RAGE mouse monoclonal antibody. Fluorescence confocal microscopic images of the cells were acquired using excitation/emission wavelengths of 590/670 nm for the proximal ligation assay (PLA) signal and 410/470 nm for the Hoechst 33342 dye (Sigma-Aldrich). Images were acquired using a confocal TCS SP8 X microscope (Leica, Wetzlar, Germany).

### Immunoblotting

Tumor tissues harvested from control (SA-treated) and hyperacetylated (ASA + TSA-treated) mice were suspended in PBS and homogenized using a sonicator (Hielscher, Teltow, Germany). Immunoblotting was performed as described previously^12^. Antibodies against RAGE (A11) and Bcl-2 (C-2) were from Santa Cruz Biotechnology. Anti-β-actin (AC-74) antibodies were from Sigma-Aldrich. Antibodies against caspase-3 (Cat. no. 9662) and acetyl-lysine (Cat. no. 9441) were from Cell Signaling Technology (Danvers, MA, USA). Antibodies against poly-ADP-ribose polymerase-1 (PARP-1) (C-2–10) and Bax (6A7) were from BD Biosciences (Bedford, MA, USA).

### Animal experiments

Female 8-week old BALB/c nude mice were purchased from Chung-Ang Laboratory Animal (Seoul, Korea). Animals were housed at 24 °C with a 12-h day/night cycle under specific pathogen-free conditions and had *ad libitum* access to a γ-ray-irradiated laboratory rodent diet (Purina Korea) and autoclaved water. All experiments were performed in accordance with relevant guidelines and regulations of the animal care unit at Chungnam National University. The animal protocols for these experiments were approved by the Ethics Committee of Animal Experimentation of Chungnam National University (No.CNUH-016-A0015).

The MDA-MB-231, BT-549, MDA-MB-468, MDA-MB-231- RAGE^OV^, MDA-MB-231-RAGE^KD^, and MDA-MB-231-FLuc TNBC cell lines were used to generate orthotropic xenografts. Mice were anesthetized, and a 5-mm skin incision was made just below the fourth nipple. TNBC cells (1.5 × 10^6^ cells/mouse; 1:1 ratio with Matrigel) were unilaterally injected into the mammary fat pad. The mice were divided into groups of six to seven and orally administered 20 mg ASA/(kg·day), and tumors were subcutaneously injected with 0.5 mg/kg TSA three times per week. Some mice were treated with 10 mg ASA/(kg·day) but were excluded from further study due to the lack of inhibitory effects on the growth and development of tumors. Control mice were treated with 0.1 ml SA using a similar dosing schedule. Body weight was recorded periodically. Tumor dimensions were measured using a caliper, and tumor volumes were estimated as follows: tumor volume = (length × width^2^) × π/6^49,50^. At the experimental endpoint, tumor tissues were harvested and used for histological and immunoblot analyses. All animal experiments were repeated at least twice with similar results.

### *In vivo* imaging

At the experimental endpoint, 3D micro-computed tomography (micro-CT) images of orthotopic xenografts were acquired. A micro-CT instrument (Quantum FX; Perkin Elmer) equipped with an open multi-focus X-ray tube and a 14-bit direct amorphous silicon flat-panel detector (Varian PaxScan 2520 D/CL; Varian, Salt Lake City, UT, USA) was used for imaging. Before image acquisition, 300 μl of a contrast agent (Iomeprol; Imeron 300; Bracco Imaging Group, Germany) was injected over 40 s via the lateral tail vein under general anesthesia. Imaging was performed using a tube voltage of 90 kV (current: 200 µA) under a 360° rotation within an 18-s scan time and using continuous image acquisition at 60 frames/s, resulting in 1,000 projections.

Bioluminescence imaging was performed using an *in vivo* imaging system (IVIS) consisting of a Lumina XRMS instrument (PerkinElmer). Animals were anesthetized by inhaling 2.5% isoflurane and received weekly intraperitoneal injections of RediJect _D_-luciferin potassium salt (150 mg/kg) or monthly intraperitoneal injections of MMPSense750 Fast (20 nmol/kg). Images were taken and analyzed using Living Image software (Caliper Life Sciences, Street Waltham, MA, USA). A luminescent camera was set to obtain images every 30 s with medium binning and 1 f/stop, and a blocked excitation and open emission filter. Photons emitted from live mice were acquired as relative luminescence units (proportional to the number of photons detected in the pixel) and as photons per s/[cm^2^· steradian (sr)].

### Measurement of plasma APE1/Ref-1

Cell-free plasma was obtained from the blood of each xenograft tumor by two centrifugation steps at 4000 × *g* at room temperature (15 min and then 5 min). The level of APE1/Ref-1 in each sample was quantified by ELISA as previously described^23^, with some modification. Briefly, 96-microwell plates (Thermo Fisher Scientific, Waltham, MA, USA) were pre-coated overnight with a mouse anti-APE1/Ref-1 antibody. After washing and blocking, a plasma sample, standard, or blank was added to each well. Plates were incubated at 37 °C for 90 min, washed, and incubated with a horseradish peroxidase (HRP)-conjugated rabbit anti-APE1/Ref-1 antibody at room temperature for 2 h. The plates were washed and tetramethyl benzidine was added to the wells. Reactions associated with color development were stopped, and the absorbance was measured at 450 nm using an automatic microtiter plate reader (Promega, Madison, WI, USA). Each sample was assayed in duplicate, and mean values were determined.

### Histologic analysis of apoptotic bodies

Tumor tissues from control and hyperacetylated xenografts were fixed in 4% paraformaldehyde, dehydrated, paraffin-embedded, and sectioned at 5 μm. Apoptosis in sections was visualized by terminal deoxyribonucleotidyl transferase-meditated dUTP nick end labeling (TUNEL) assays using the ApopTag Plus peroxidase *in situ* apoptosis kit (EMD Millipore, Billerica, MA, USA).

### Immunohistochemical analyses

Fixed tumor tissues were dehydrated, paraffin-embedded, and sectioned at 5 μm. After quenching endogenous peroxidase with 3% hydrogen peroxide for 15 min, sections were treated with normal horse serum. The sections were then incubated overnight at 4 °C with primary anti-proliferating cell nuclear antigen (PCNA) (PC 10; Sigma-Aldrich), anti-CD31 (polyclonal; Abcam, Cambridge, UK), anti-MMP-9 (D6O3H; Cell Signaling Technology) and anti-acetyl-lysine (polyclonal; Cell Signaling Technology) antibodies. HRP-conjugated anti-rabbit or anti-mouse IgG was next applied for 60 min at room temperature. Color was developed for 3 min by incubation with 3,3′-diaminobenzidine (DakoCytomation, Santa Clara, CA, USA). Sections were counterstained with hematoxylin and eosin (H&E) and examined under a Motic microscope (Motic, Richmond, BC, Canada) at 40× magnification.

### Reactive oxygen species measurement

Fresh tumor samples were fixed in an acetone solution. Frozen tissues were cut into 30-μm sections using a cryostat, washed, and treated with dihydroethidium (DHE) (Thermo Fisher Scientific). Sections were incubated for 7 min at 37  °C in the dark and washed twice with PBS. Images were taken immediately after mounting with Vectashield containing 4′,6-diamidino-2-phenylindole (Sigma-Aldrich). The fluorescence intensity of at least 100 nuclei per sample was scored in at least two mice per experimental condition.

### Cell viability and apoptotic DNA fragmentation assay

The effect of recombinant human Ac-APE1/Ref-1 on the cell viability and DNA fragmentation of MDA-MB-231, MDA-MB-468, BT-549, MCF-7 cells, HMECs, and HUVECs was determined using the RealTime-Glo MT luminescent kit (Promega, Madison, WI, USA) and Cell Death Detection ELISA kit (Roche Applied Science, IN, USA), respectively. Acetylated recombinant human Ac-APE1/Ref-1 was prepared as previously described^12^.

### Statistical analyses

Group means were compared using unpaired *t*-test or one-way ANOVA followed by Dunnett’s or Bonferroni’s multiple-comparison test with GraphPad Prism version 5.01 (GraphPad Software, Inc., San Diego, CA, USA). *P* < 0.05 was considered significant.

### Data availability

All data generated or analysed during this study are included in this published article (and its Supplementary Information files).

## Electronic supplementary material

Supplementary data
