# Supplementary material for: Therapeutic positioning of secretory acetylated APE1/Ref-1 requirement for suppression of tumor growth in triple-negative breast cancer in vivo
Source: Sci Rep. 2018 Jun 7;8:8701. doi: 10.1038/s41598-018-27025-9 (PMC5992149; doi:10.1038/s41598-018-27025-9)
Supplement: Supplementary file 1 — Supplementary data [file 41598_2018_27025_MOESM1_ESM.doc]

**Therapeutic positioning of secretory acetylated APE1/Ref-1 requirement for suppression of tumor growth in triple-negative breast cancer *in vivo***

**Yu Ran Lee^1^, Myoung Soo Park^2^**, **Hee Kyoung Joo^1^, Ki Mo Kim^3^,** **Jeryong Kim^4^, Byeong Hwa Jeon^1*^, Sunga Choi^1*^**

**Author’s Affiliations:** ^1^Research Institute of Medical Sciences, Department of Physiology, School of Medicine, Chungnam National University, Daejeon, 35015; ^2^Preclinical Research Center, Chungnam National University Hospital, Daejeon 35015; ^3^Korean Medicine Convergence Research Division, Korea Institute of Oriental Medicine (KIOM), Daejeon, 34054; ^4^Department of Surgery, School of Medicine, Chungnam National University, Daejeon, 35015

***Address correspondence to:**

Byeong Hwa Jeon, M.D., Ph.D., Phone: 82-42-580-8214; Fax: 82-42-585-8440; E-mail: bhjeon@cnu.ac.kr; Department of Physiology, School of Medicine, Chungnam National University, 266 Munhwa-ro, Jung-gu, Daejeon, 35015, Korea.

Sunga Choi, Ph.D., Phone: 82-42-280-6767; Fax: 82-42-280-6767; E-mail: sachoi@cnu.ac.kr; Department of Physiology, School of Medicine, Chungnam National University, 266 Munhwa-ro, Jung-gu, Daejeon, 35015, Korea.

**
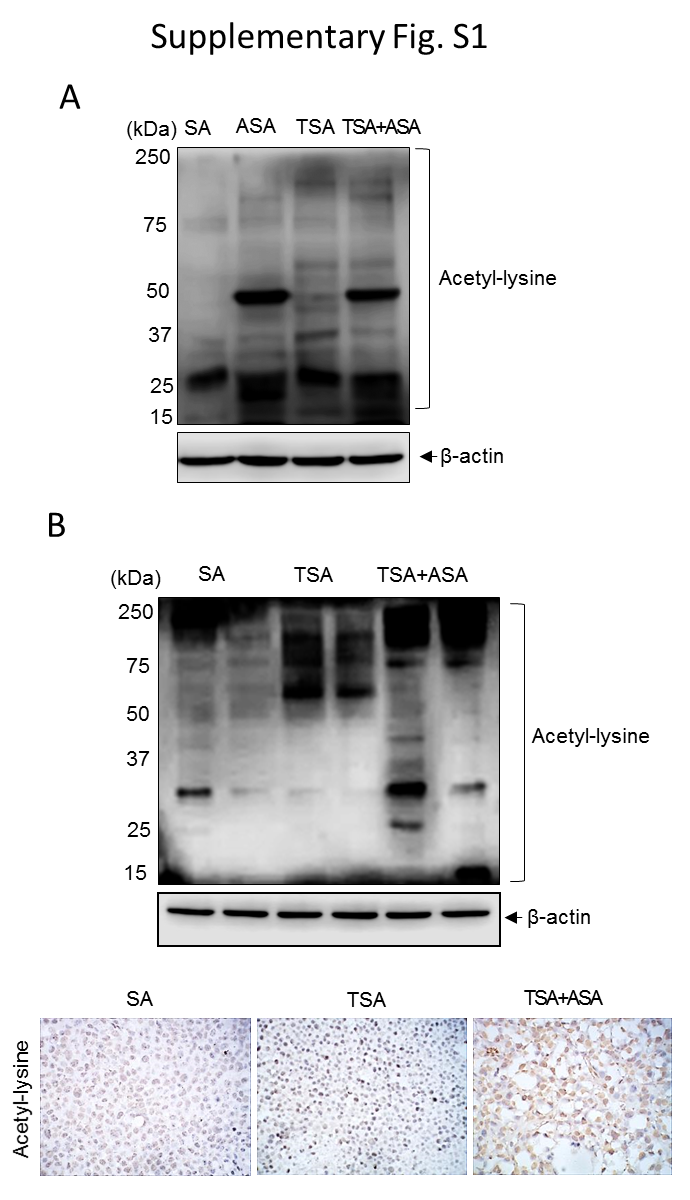
**

**Supplementary Figure 1** Acetylation of intracellular proteins. A and B , Cell lysates or tumor tissues were obtained from MDA-MB-231 cells treated with SA, 5 mM ASA, 1 𝛍M TSA, or 0.1 𝛍M TSA+5mM ASA or from MDA-MB-231 xenografts treated three times weekly with 20 mg/(kg•day) ASA plus subcutaneous injection of 0.5 mg/kg TSA, TSA alone, or SA. Cellular proteins and tumors tissues underwent immunoblotting and immunohistochemistry using the anti-acetyl lysine antibody. The blots were stripped and reprobed with a β-actin antibody to ensure equal protein loading. Immunoblotting for each protein was performed two or more times by using independently prepared lysates with similar results.

**
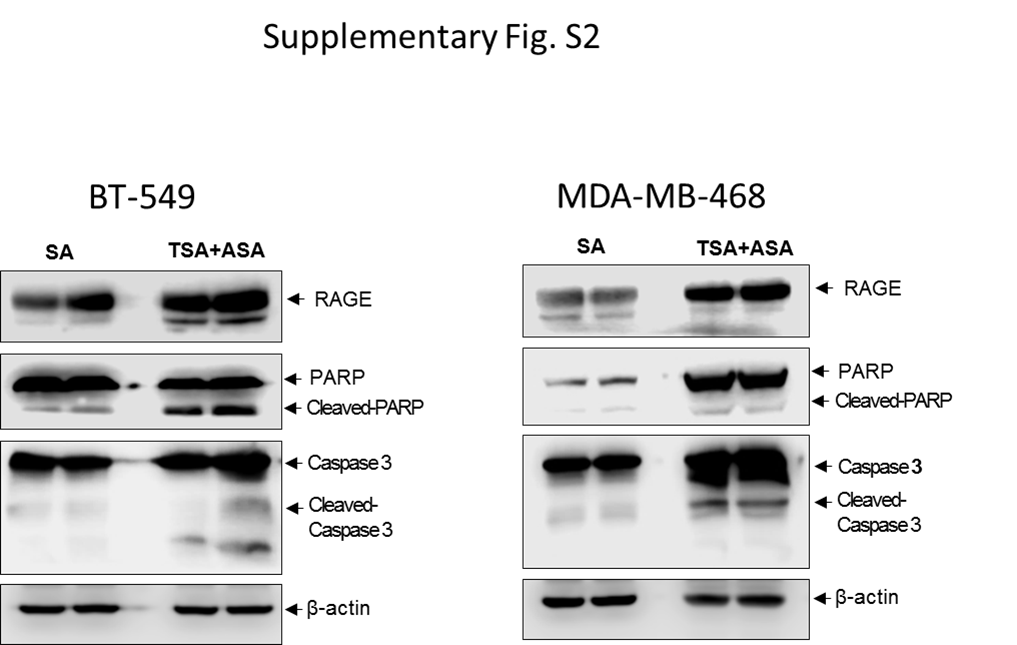
**

**Supplementary Figure 2** Hyperacetylation induces apoptosis in BT-549, and MDA-MB-468 xenografts. Immunological analysis of the tumor tissues from BT-549 and MDA-MB-468 xenografts treated with 20 mg/(kg•day) ASA plus subcutaneous injection of 0.5 mg/kg TSA or SA (as a negative control) three times weekly. Representative immunoblots showing RAGE, caspase-3, and PARP-1 expression. The blots were stripped and reprobed with a β-actin antibody to ensure equal protein loading. Immunoblotting for each protein was performed two or more times by using independently prepared lysates with similar results.


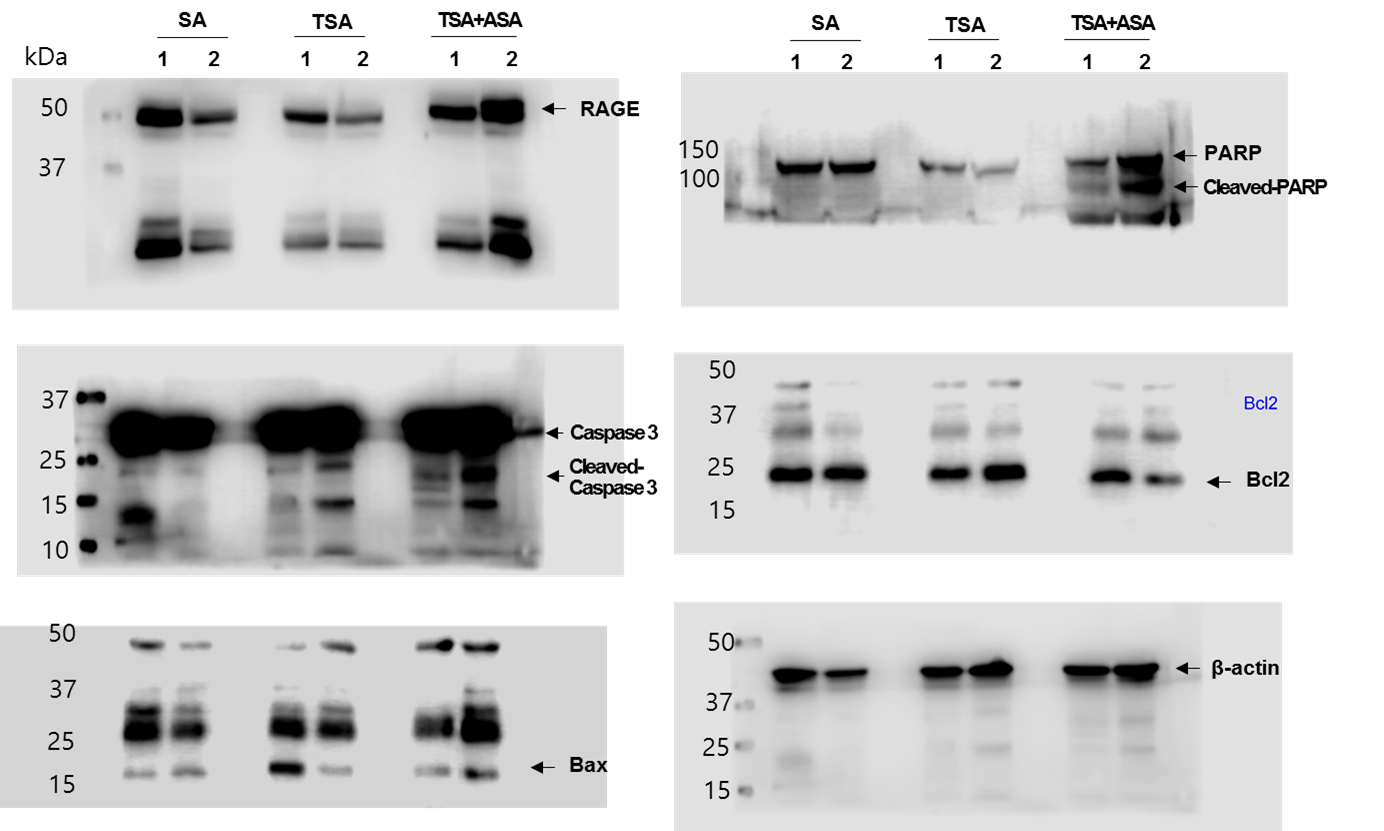


**Supplementary Figure 3** Immunological analysis of the tumor tissues from hyperacetylated MDA-MB-231 cell xenografts treated with 20 mg/(kg•day) ASA plus subcutaneous injection of 0.5 mg/kg TSA or SA (as a negative control) three times weekly. Full-length blots showing RAGE, Bax, Bcl-2, and caspase-3, PARP-1 expression and β-actin as a protein-loading control.

**
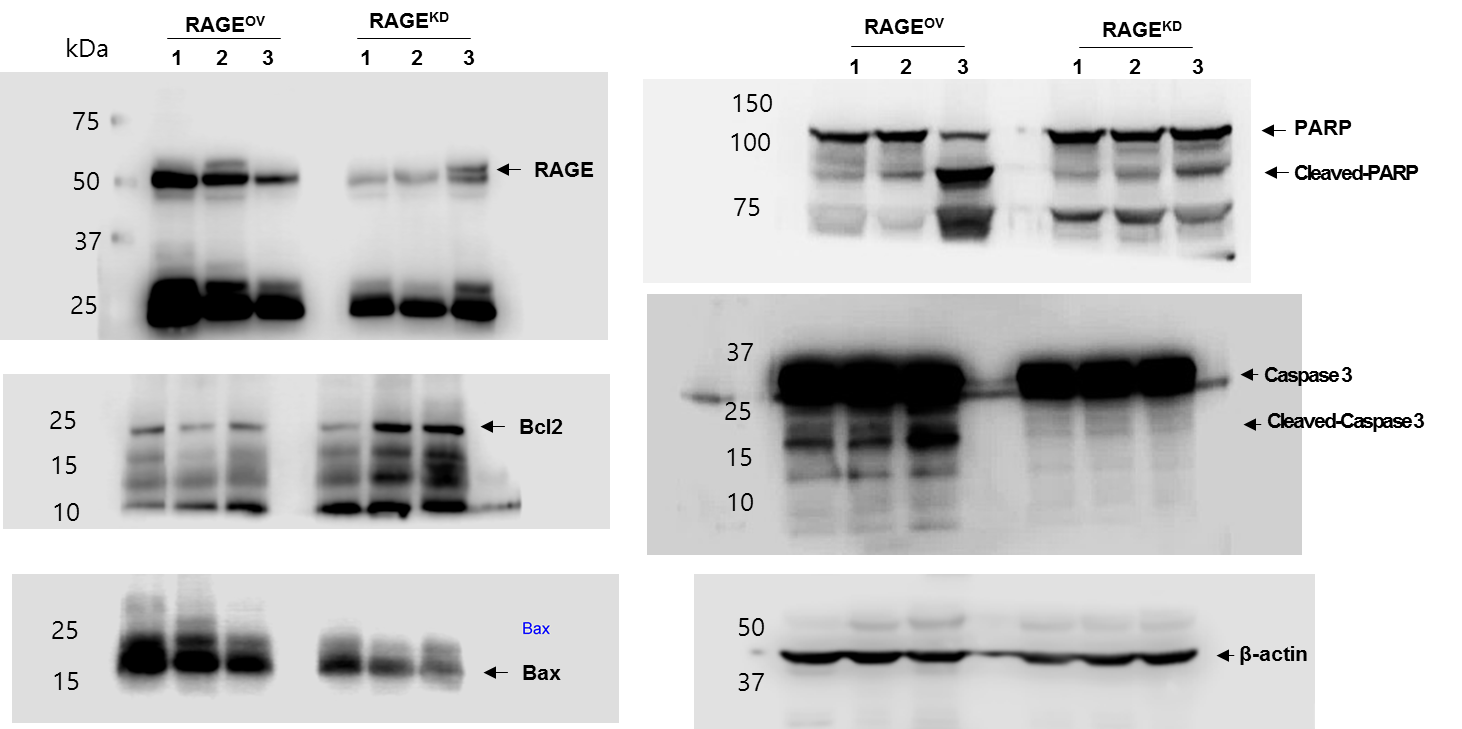
**

**Supplementary Figure 4** Immunological analysis of the tumor tissues from hyperacetylated xenografts implanted with MDA-MB-231 cells overexpressing RAGE (RAGE^OV^) or with knocked-down RAGE expression (RAGE^KD^). Xenografts treated with 20 mg/(kg•day) ASA plus subcutaneous injection of 0.5 mg/kg TSA three times weekly. Full-length blots showing RAGE, Bax, Bcl-2, and caspase-3, PARP-1 expression and β-actin as a protein-loading control.
